# Enzymatic Depletion of Mitochondrial Inorganic Polyphosphate (polyP) Increases the Generation of Reactive Oxygen Species (ROS) and the Activity of the Pentose Phosphate Pathway (PPP) in Mammalian Cells

**DOI:** 10.3390/antiox11040685

**Published:** 2022-03-31

**Authors:** Vedangi Hambardikar, Mariona Guitart-Mampel, Ernest R. Scoma, Pedro Urquiza, Gowda G. A. Nagana, Daniel Raftery, John A. Collins, Maria E. Solesio

**Affiliations:** 1 Department of Biology and Center for Computational and Integrative Biology (CCIB), College of Arts and Sciences, Rutgers University, Camden, NJ 08103, USA; vdh15@rutgers.edu (V.H.); mg1616@camden.rutgers.edu (M.G.-M.); ers115@camden.rutgers.edu (E.R.S.); pu23@camden.rutgers.edu (P.U.); 2 Mitochondrial and Metabolism Center, University of Washington, Seattle, WA 98109, USA; ngowda@uw.edu (G.G.A.N.); draftery@uw.edu (D.R.); 3 Department of Orthopedic Surgery, Sidney Kimmel Medical College, Thomas Jefferson University, Philadelphia, PA 19107, USA; john.collins2@jefferson.edu

**Keywords:** mitochondria, mitochondrial inorganic polyphosphate, polyP, ROS, antioxidants, pentose phosphate pathway, mammalian bioenergetics

## Abstract

Inorganic polyphosphate (polyP) is an ancient biopolymer that is well preserved throughout evolution and present in all studied organisms. In mammals, it shows a high co-localization with mitochondria, and it has been demonstrated to be involved in the homeostasis of key processes within the organelle, including mitochondrial bioenergetics. However, the exact extent of the effects of polyP on the regulation of cellular bioenergetics, as well as the mechanisms explaining these effects, still remain poorly understood. Here, using HEK293 mammalian cells under Wild-type (Wt) and MitoPPX (cells enzymatically depleted of mitochondrial polyP) conditions, we show that depletion of polyP within mitochondria increased oxidative stress conditions. This is characterized by enhanced mitochondrial O_2_^−^ and intracellular H_2_O_2_ levels, which may be a consequence of the dysregulation of oxidative phosphorylation (OXPHOS) that we have demonstrated in MitoPPX cells in our previous work. These findings were associated with an increase in basal peroxiredoxin-1 (Prx1), superoxide dismutase-2 (SOD2), and thioredoxin (Trx) antioxidant protein levels. Using ^13^C-NMR and immunoblotting, we assayed the status of glycolysis and the pentose phosphate pathway (PPP) in Wt and MitoPPX cells. Our results show that MitoPPX cells display a significant increase in the activity of the PPP and an increase in the protein levels of transaldolase (TAL), which is a crucial component of the non-oxidative phase of the PPP and is involved in the regulation of oxidative stress. In addition, we observed a trend towards increased glycolysis in MitoPPX cells, which corroborates our prior work. Here, for the first time, we show the crucial role played by mitochondrial polyP in the regulation of mammalian redox homeostasis. Moreover, we demonstrate a significant effect of mitochondrial polyP on the regulation of global cellular bioenergetics in these cells.

## 1. Introduction

Inorganic polyphosphate (polyP) is an ancient and ubiquitous molecule. It has been exceptionally well-conserved throughout evolution, and it is present in all studied organisms, including mammals [1,2]. This highly negatively charged and multifunctional polymer is composed of multiple orthophosphates bound together by high-energy phosphoanhydride bonds, similar to those found in ATP [2,3,4]. In fact, the rapid inter-conversion of phosphoryl groups between ATP and polyP has already been demonstrated [5]. In mammalian cells, polyP is usually comprised of a few hundred units of orthophosphate, and concentrations are within the micromolar range [6,7]. Interestingly, the late Nobel Laureate Dr. A. Kornberg, who devoted the final years of his career to the study of polyP, reported that bacteria with decreased levels of polyP show increased sensitivity to diverse stressors, including specific chemicals and heat shock [2,8]. Recently, the crucial role played by polyP in the regulation of bioenergetics in various organisms, including mammals, has been demonstrated by ourselves and others [3,9,10,11,12,13,14,15,16,17,18,19,20]. Moreover, different authors have demonstrated that polyP is involved in the regulation of many other physiological processes, which are also closely related to bioenergetics (Table 1). However, the exact extent of these effects, as well as the mechanism(s) underlying them, remain poorly understood [3,9,10,11,12,13,14,15,16,17,18,19].

Mammalian bioenergetics is a complex set of processes. It encompasses diverse mechanisms by which the demand and production of cellular ATP are matched and scaled to meet the varying metabolic needs of the cell at each precise moment. These mechanisms include, but are not limited to, oxidative phosphorylation (OXPHOS), glycolysis, and the Pentose Phosphate Pathway (PPP). Mammalian bioenergetics is also closely related to the regulation and the production of reactive oxygen species (ROS). For example, the vast majority of ROS and its precursors in mammalian cells are produced in the electron transfer chain (ETC) due to incomplete or dysfunctional OXPHOS [31,32]. Accordingly, when OXPHOS is dysregulated, increased ROS are present in the cell, which contribute to enhanced oxidative stress conditions which can lead to non-discriminatory damage to proteins, lipids, and DNA, act as a transducer of catabolic redox signaling events, and promote apoptotic cell death [33]. To counteract the increased generation of ROS and prevent oxidative stress, mammalian cells contain a suite of antioxidant systems that are responsible for the detoxification of ROS, including peroxiredoxins, thioredoxins, superoxide dismutases, and catalases [34,35,36,37]. Moreover, the mammalian antioxidant system is partially regulated by NADPH, which is mainly generated by the PPP since NADPH is the donor of reductive potential to several antioxidant systems [38]. For example, NADPH derived from the PPP is necessary for the reduction in oxidized glutathione (GSSG) to its active form of reduced glutathione (GSH) [39,40]. The PPP is in tight equilibrium with glycolysis [39], and both processes are closely related to the status of OXPHOS.

We have recently demonstrated the important regulatory effects of mitochondrial polyP on mammalian OXPHOS and glycolysis in vitro [17]. Specifically, we showed decreased OXPHOS and increased glycolysis in cells enzymatically depleted of mitochondrial polyP (MitoPPX), but we did not address the status of the PPP in our samples. Interestingly, due to the potent regulatory effects of polyP on mammalian bioenergetics, polyP could also play a role in the maintenance of redox homeostasis in these cells. In fact, the protective effects of polyP against the increased generation of ROS have been described in bacteria [41,42]. Moreover, recent data demonstrate that cells with altered levels of polyP, due to a modification of the enzyme Nudt3, display an increase in oxidative stress levels, DNA damage, and enhanced cell death [43]. However, little is known about the exact regulatory role of polyP on ROS generation in mammalian cells.

The metabolism of polyP is relatively well described in yeast and bacteria [44,45,46,47,48,49,50,51,52,53,54], but it remains poorly understood in mammalian cells. A recently published study established that the mammalian mitochondrial F_0_F_1_ ATPase can be stimulated by polyP, and that this enzyme is involved in the metabolism of the polymer [55]. Since knocking down the mammalian F_0_F_1_ ATPase is not feasible due to its direct effects on cell viability, we have created HEK293 cells enzymatically-depleted of mitochondrial polyP (MitoPPX cells) [9,10]. Here, using Wild-type (Wt) and MitoPPX cells, we expand on our previous study to demonstrate that the loss of mitochondrial polyP significantly increases the generation of O_2_^−^ and H_2_O_2_, an effect that is associated with an increase in multiple antioxidant protein levels. Additionally, we show that the PPP is upregulated in response to the depletion of mitochondrial polyP. Lastly, our data show that the levels of the enzyme transaldolase (TAL), which is a key rate-limiting enzyme of the non-oxidative phase of the PPP, are increased in MitoPPX cells when compared to Wt controls. Similarly to polyP, TAL is also well-conserved throughout evolution [56], and it plays an important role in the regulation of cellular ROS [56].

Elucidating the exact regulatory role of mitochondrial polyP on cellular bioenergetics could lead to the development of novel pharmacological and therapeutic strategies aimed at targeting oxidative stress and dysregulated bioenergetics, which represent two key features in the etiopathogenesis of some of the main aging-associated diseases, such as neurodegenerative disorders. In fact, dysregulated bioenergetics, including alterations in glycolysis and the PPP, have been broadly described in a wide variety of conditions and pathologies, including Parkinson’s disease and Alzheimer’s disease [57,58,59,60].

## 2. Materials and Methods

### 2.1. Reagents

Dulbecco’s Modified Eagle medium (DMEM), DMEM no glucose, penicillin–streptomycin, Hank’s Balanced Salt Solution (HBSS), G418 (Geneticin), trypsin, and heat-inactivated fetal bovine serum (FBS) were purchased from Gibco-Invitrogen (Carlsbad, CA, USA). Phosphate-Buffered Saline (PBS), poly-L-lysine, Tween-20, Dimethyl sulfoxide (DMSO), 2-vinyl pyridine, D-Glucose-1,2-^13^C_2_, Methanol, MTT, trimethylsilylpropionic acid-d_4_, and sodium salt were purchased from Sigma-Aldrich (St. Louis, MI, USA). In addition, 4′-6-diamino-2-phenylindole (DAPI), Pierce BCA Protein Assay kit, and Pierce ECL Western Blotting Substrate were purchased from Thermo Fisher Scientific (Waltham, MA, USA). Amplex Red Hydrogen Peroxide/Peroxidase Assay Kits and Glutathione Colorimetric Detection Kits were purchased from Invitrogen (Carlsbad, CA, USA). Secondary antibodies, trypan blue, and all the materials and reagents used in the immunoblotting experiments were obtained from BioRad (Hercules, CA, USA). All the anti-rabbit secondary antibodies were used at a 1:1000 dilution, while anti-mouse secondary antibodies were used at 1:2000 dilution. ATP luminescent measurement kits were purchased from Abcam (Cambridge, Cambridgeshire, UK); PolyP standards were a gift from Dr. Toshikazu Shiba, from Kitasato University, Tokyo, Japan. The following antibodies were used for immunoblotting: anti-G6PD (anti-rabbit, Sigma-Aldrich, HPA000247), anti-PGD (anti-rabbit, Sigma-Aldrich, HPA031314), anti-TK (anti-mouse, Santa Cruz Biotechnology, sc-390179), anti-TAL (anti-mouse, Santa Cruz Biotechnology, sc-166230), anti-MnSOD (anti-rabbit, Millipore Sigma, 06-984-25UG), anti-Prx1 (anti-mouse, Abcam, ab106834), Anti-Trx (anti-rabbit, Cell Signaling technology, 2429), anti-Nrx (anti-rabbit, Proteintech, 16128-1-AP), anti-Prx3 (anti-rabbit, Abcam, ab73349), anti-Nrf2 (anti-rabbit, Abcam, ab137550), and anti-β-actin (anti-mouse, Abcam, ab8226). Anti-rabbit and anti-mouse secondary antibodies (1706516 and 1706515, respectively) were purchased from BioRad (Hercules, CA, USA).

### 2.2. Cell Culture

HEK293 cells were obtained from the American Type Culture Collection (ATCC, Manassas, VA, USA). MitoPPX cells (cells enzymatically depleted of mitochondrial polyP) were created by stable transfection of HEK293 cells with a mammalian construct containing a mitochondrial sequence for the expression of the yeast exopolyphosphatase enzyme, as we have previously described [9,10]. Wt and MitoPPX cell cultures were grown following the instructions of the ATCC. Specifically, we used DMEM supplemented with 10% (*v*/*v*) FBS and 1% penicillin-streptomycin. In the case of the MitoPPX cells, geneticin was added as the selection antibiotic (40 μg/mL) [61]. Cells were maintained in a humidified incubator at 37 °C and 5% CO_2_ atmosphere.

### 2.3. Cell Viability Assay and Cell Growth Curve

To assay cell viability in Wt and MitoPPX HEK293 cells, we used the MTT (3-(4,5-dimethylthiazol-2-yl)-2,5-diphenyletrazolium bromide) assay. Specifically, we plated 100,000 cells per well on a 96-well plate. After 24 h, we replaced the growing medium with fresh medium containing 5 mg of MTT per each 10-mL medium. Cells were then incubated for one hour in the humidified incubator. Subsequently, the medium containing MTT was replaced by DMSO, the plate was gently shaken, and absorbance was measured at 540 nm (alive cells) and 685 nm (background). Background was subtracted from the value obtained from the measurement of alive cells. The experiment was conducted in triplicate and experiments were conducted on three independent days.

To corroborate the cell viability results and to assay cell growth of the HEK293 Wt and MitoPPX cells, we used trypan blue exclusion assay [62], and quantified cell viability. Using this method and an automatic cell counter (TC20, Bio Rad, Hercules, CA, USA), we quantified the number of cells that were alive and dead. For these experiments, 300,000 HEK293 Wt and MitoPPX cells were plated in six-well culture plates. In the case of the cell viability assay, it was assayed 48 h later, while, to conduct the growth curve, cells were collected 24 h, 48 h, and 72 h after seeding. Cells were collected for the assay by trypsinization and the experiment was conducted following the indications of the manufacturer. Experiments were conducted in triplicate.

### 2.4. Cell Imaging

First, 100,000 cells/well were plated in six-well plates. After 48 h, cells were incubated in the dark for 15 min in the presence of 20 μM DAPI. Subsequently, cells were imaged using transmitted light and a DAPI filter on an EVOS Cell Imaging System (Life Technologies, Carlsbad, CA, USA). The contract and brightness of the images were then adjusted using ImageJ software (NIH, Bethesda, MD, USA).

### 2.5. Quantification of Mitochondrial polyP

Mitochondria from Wt and MitoPPX cells were isolated as previously described [63,64,65]. After that, mitochondria-enriched fractions were lysed by three freeze/thaw cycles of 30 min each, at −80 °C and room temperature. To standardize the samples, protein quantification was performed using BCA assay. Next, 20 µg of protein per sample was loaded into a 96-well plate in triplicate and incubated with 10 µM of DAPI. PolyP shifts the fluorescence emission of associated DAPI towards higher wavelengths (λ_ex_ = 405 nM) [6,56,57]. After 15 min of incubation in the dark and at room temperature, plates were read at 525 nm, with intermittent agitation after 20 s, using a BioTek spectrophotometer (Thermo Fisher Scientific, Waltham, MA, USA). Synthetic polyP was used to prepare the standards for each assay. Concentration of polyP in the samples was extrapolated from the standard curve readings.

### 2.6. Measurement of ATP Levels

Cellular levels of ATP were measured using the Luminescent ATP Detection Kit (ab113849, AbCam, Cambridge, Cambridgeshire, UK) following the protocol indicated by the manufacturer. Briefly, 10,000 Wt and MitoPPX cells per well were plated in a white 96-well plate. After a 48-h incubation period, cells were lysed and luminescence was measured using a BioTek spectrophotometer (Thermo Fisher Scientific, Waltham, MA, USA). Each experiment consisted of three technical replicates. Moreover, experiments were conducted a minimum of three times on separate days.

### 2.7. Quantification of Mitochondrial O_2_^−^ Levels

Mitochondrial O_2_^−^ levels were measured in live cells using the MitoSOX Red mitochondrial O_2_^−^ indicator. Cells were plated 48 h prior to the experiment in sterile, poly-lysine treated coverslips. On the day of the experiment, the coverslips containing the cells were incubated for 10 min with 5 μM MitoSOX which was previously dissolved in 1× HBSS, following the manufacturer’s protocol (MitoSOX, M36008, Invitrogen, Waltham, MA, USA). Subsequently, fluorescence was visualized by confocal microscopy and a 40× oil-immersion objective (SP8 Leica, Wetzlar, Germany). Five images per cover slip were captured from biological replicates. Quantification of fluorescence intensity was performed using ImageJ software (NIH, Bethesda, MD, USA).

### 2.8. Quantification of Intracellular H_2_O_2_ Levels

Intracellular H_2_O_2_ levels were assayed using Amplex Red Hydrogen Peroxide/Peroxidase Assay kit, following the manufacturer’s instructions. In the presence of peroxidase, the Amplex Red reagent reacts with H_2_O_2_ in a 1:1 stoichiometry to form resorufin, which is a red-fluorescent oxidation product. Wt and MitoPPX cells were plated 48 h before the assay and collected to be lysed in a detergent-free lysis buffer. Lysed samples, normalized by protein content, were added in triplicate to a 96-well plate. Amplex Red was subsequently added to each well and the mixture was incubated for 30 min at room temperature. Fluorescence was then measured at λ_ex_ 530–560 nm and λ_em_ 590 nm, using a BioTek spectrophotometer (Thermo Fisher Scientific, Waltham, MA, USA). Control conditions were performed using blank readings obtained from wells containing culture media and assay reagents, in the absence of cells, for each experiment. After background correction, the obtained fluorescence values were normalized to the mean of the Wt control fluorescence value. Every experiment included samples in triplicate, and the assay was repeated on at least three independent days.

### 2.9. Preparation of the Cell Samples for NMR Analysis

To begin, 5 × 10^6^ cells were seeded in p150 cell culture-treated plates for 24 h. Cells were then treated with 4.5 mM D-glucose-1,2-^13^C_2_ in glucose-free media DMEM for additional 24 h. Subsequently, cells were harvested with ice cold 1× PBS and stored at −80 °C prior to analysis.

### 2.10. NMR Analysis of the PPP

For extraction of metabolites, cellular pellets were mixed with methanol (800 μL), vortexed for 60 s, and added with deionized water (400 μL). The solution was vortexed again for 60 s and sonicated for 20 min on an ice bath. Subsequently, the solutions were vortexed again for 60 s and centrifuged at 14,000× *g* for 30 min. The supernatant solutions that contained water soluble metabolites were transferred to clean 2 mL Eppendorf tubes and dried using nitrogen gas. Each dried residue was then dissolved in 200 μL phosphate buffer prepared in deuterium oxide solvent (100 mM, pH 7.4) by dissolving 1124.0 mg anhydrous Na_2_HPO_4_ and 249.9 mg anhydrous NaH_2_PO_4_ in 100 g D_2_O; sodium salt of trimethylsilylpropionic acid-d_4_ (TSP, 4 mM) was added to the buffer to serve as the chemical shift reference. The solutions were centrifuged for 1 min to sediment any particulate matter and the supernatants were transferred to 3 mm NMR tubes for analysis. All chemicals were obtained from Sigma-Aldrich (St. Louis, MI, USA).

Using a Bruker AVANCE III 800 MHz spectrometer (Billerica, MA, USA) equipped with a cryoprobe at ^13^C frequency of 201.21 MHz, ^13^C NMR spectra were obtained at 298 K temperature. One-dimensional NMR spectra were obtained using a one pulse sequence with a power-gated decoupling of ^1^H nuclei using WALTZ-16 pulse sequence. For each sample, 128,000 data points were acquired using a spectral width of 42,613 Hz, a relaxation delay of 1.5 s, and 16,000 or 38,000 scans. The data were processed using a spectral size of 131,072 points and by multiplying with an exponential window function using a line broadening of 1.0 Hz. The spectra were phase and baseline corrected and referenced with respect to the ^13^C signal from the reference compound, TSP. Bruker Topspin version 4.1.0 software package was used for NMR data acquisition and processing.

Quantitation of glucose flux through glycolysis and the PPP was based on lactate peaks from the ^13^C NMR spectra, as lactate is an end product of both glycolysis and the PPP. Treating cells with [1,2-^13^C_2_]-glucose enables distinguishing between lactate produced by the glycolysis and that produced by the PPP [66]; glucose flux through glycolysis yields [2,3-^13^C_2_]-lactate as an end product, while the flux through the PPP yields [3-^13^C_1_]-lactate as an end product. Therefore, the area of the doublet peak from lactate C_3_ (22.928 ppm) provided glucose flux through glycolysis, whereas the area of the single peak arising from the same carbon provided the glucose flux through the PPP. The single peak area of lactate C_3_ was corrected for the contribution from natural abundance ^13^C based on the singlet peak area of lactate C_2_ (71.348 ppm), after accounting for the difference in nuclear Overhauser enhancement between lactate C_2_ and lactate C_3_. The Bruker Topspin version 4.1.0 software package was used for integration of the ^13^C NMR peaks.

### 2.11. Immunoblotting Assays

First, 2.5 × 10^6^ cells were seeded in a Petri dish. Then, 48 h later, cells were harvested and lysed at 4 °C for 30 min under gentle agitation in a standard lysis buffer containing inhibitors of proteases and phosphatases [67]. Subsequently, the samples were centrifuged at 12,000× *g* for 10 min to pellet insoluble proteins. The supernatants were collected and protein was quantified using the BCA assay. Immunoblotting assays were conducted on the cell lysates under reducing conditions, using 20 μg of protein and the specified antibodies by following the protocol that we have previously used [67,68,69,70,71]. In all cases, β-actin was used as a loading control. Primary antibodies were used at a 1:1000 dilution unless otherwise stated. Densitometric analysis was performed using ImageJ software and Graphpad Prism version 9 (NIH, Bethesda, MD, USA; San Diego, CA, USA; respectively).

### 2.12. Statistical Analysis

All data are presented as mean ± SD. Statistical significance of the differences between groups was determined by Student’s t-test. The level of statistical significance was set at α = 0.05 (* *p* ≤ 0.05, ** *p* ≤ 0.01, *** *p* ≤ 0.001). For the statistical analysis and graphical representation, OriginLab software and GraphPad Prism version 9 were used (Northampton, MA, USA; San Diego, CA, USA; respectively).

## 3. Results

### 3.1. Isolated Mitochondria from MitoPPX Cells Show Decreased Levels of polyP

We have already validated and utilized stable HEK293 MitoPPX cells [9,10]. Moreover, our previous study demonstrates dysregulated OXPHOS, increased glycolysis, and decreased ATP production in response to depletion of mitochondrial polyP, using the same cellular model that we have used in this study [17]. Here, we first corroborated our prior findings and observed a significant decrease in ATP levels in MitoPPX cells, as compared to Wt cells (Supplementary Figure S1A), as well as increased levels of glycolysis in our mutant samples (Supplementary Figure S1B). These findings corroborate our previous data and demonstrate that the HEK293 MitoPPX cells used in this paper have the same bioenergetic phenotype as the one used in our previous work [17].

**Figure 1:**
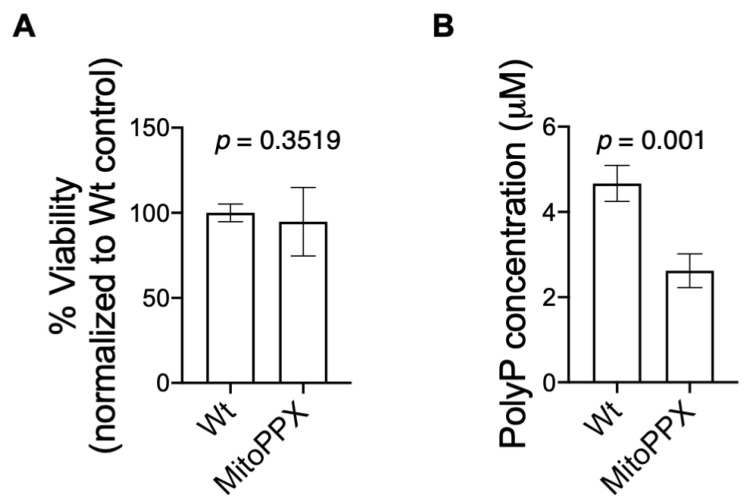
Mitochondria isolated from MitoPPX cells show decreased levels of polyP. However, the enzymatical depletion of the polymer within the organelle does not have a deleterious effect on cell viability in HEK293 cells. (**A**) Graph showing the results of the MTT cell viability assays. Note that no significant differences in terms of cell viability were found between Wt and MitoPPX cells in this experiment. (**B**) Graph showing the concentration of polyP in mitochondria isolated from Wt and MitoPPX cells. To demonstrate the decreased levels of the polymer in mitochondria isolated from MitoPPX cells, polyP was measured using DAPI fluorescence after the isolation of the organelles. PolyP concentrations were determined based on extrapolation from the standard curve, which was prepared with exogenous polyP. Data in the graph are shown as mean ± standard deviation of triplicates obtained from at least three independent experiments (*n* = 3). Unpaired t-tests were used to detect significant differences between Wt and MitoPPX cells. *Abbreviations used in this figure:* MitoPPX: cells expressing the exopolyphosphatase enzyme in mitochondria; Wt: Wild-type cells; PolyP: inorganic polyphosphate, MTT: 3-(4,5-dimethylthiazol-2-yl)-2,5-dypheniyltretazolium bromide, DAPI: 4′-6-diamidino-2-phenylindole.

Our data also show that the enzymatic depletion of mitochondrial polyP does not have a deleterious effect on cell viability, measured by MTT assay and corroborated by trypan blue assay (Figure 1A and Figure S2A). Moreover, both Wt and MitoPPX cells showed a similar growth curve, specially 48 h after seeding them (Supplementary Figure S2B). This is the time at which the experiments included in this manuscript were conducted. Cell growth was assayed by measuring cell proliferation 24 h, 48 h, and 72 h after seeding the same number of Wt and MitoPPX cells. We did not observe any differences in cell morphology between Wt and MitoPPX HEK293 cells (Supplementary Figure S2C). Here, for the first time, we measured the concentration of polyP within isolated mitochondria, in both Wt and MitoPPX cells. Our data show that mitochondria isolated from MitoPPX cells display a significant reduction in PolyP (55.7%), when compared with mitochondria isolated from Wt cells (Figure 1B). Specifically, the concentration of polyP was 2.62 μM in mitochondria isolated from MitoPPX cells and 4.70 μM in mitochondria isolated from Wt samples. To quantify the amount of mitochondrial polyP, we used the measurement of the DAPI-polyP fluorescence, which is a well-established method to assay the polymer [6,16,56]. This finding aligns with our previous work, which demonstrates the increased ability of MitoPPX cells to degrade exogenous polyP, when compared with Wt cells [17].

### 3.2. MitoPPX Cells Display Increased Levels of O_2_^−^ and H_2_O_2_

**Figure 2:**
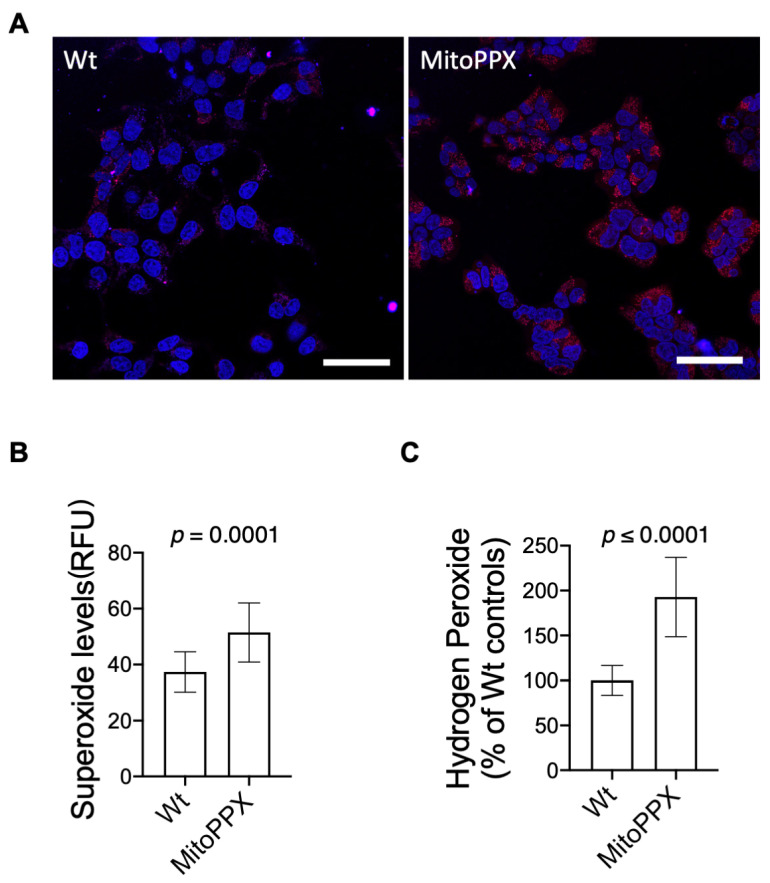
MitoPPX cells show increased levels of ROS. (**A**) Representative confocal images showing increased levels of O_2_^−^, measured by MitoSox Red, in MitoPPX cells, when compared with the Wt cells. (**B**) Quantification of the confocal images obtained after the incubation of samples with MitoSox Red. (**C**) Quantification of the fluorescence signal in Wt and MitoPPX cells after the incubation of the samples with Amplex Red to measure H_2_O_2_ generation. Data in all graphs are shown as mean ± standard deviation of at least 60 fields (MitoSox Red); triplicates were obtained from at least three independent experiments. Unpaired t-tests were used to detect significant differences between Wt and MitoPPX cells. Scale bar: 200 μM. *Abbreviations used in this figure:* ROS: reactive oxygen species; MitoPPX: cells expressing the exopolyphosphatase enzyme in mitochondria; Wt: Wild-type cells; O_2_^−^: superoxide; H_2_O_2_: hydrogen peroxide.

A well-known consequence of dysregulated OXPHOS is the increased levels of ROS. Here, we demonstrate that MitoPPX cells display a significant increase in the intracellular levels of ROS when compared to Wt cells. Specifically, using MitoSOX Red to quantify mitochondrial O_2_^−^ generation, we show that MitoPPX cells display a 37.7% increase in O_2_^−^ levels, when compared to Wt controls (*p* ≤ 0.001) (Figure 2A,B). Furthermore, using Amplex Red reagent to detect H_2_O_2_ levels, we also demonstrate that MitoPPX cells show a 92.8% increase in the levels of H_2_O_2_ when compared to Wt samples (Figure 2C). These data demonstrate that the enzymatic depletion of mitochondrial polyP significantly increases intracellular ROS levels in HEK293 cells. This effect probably occurs through the regulatory effects of polyP on OXPHOS that have already been demonstrated [17].

### 3.3. MitoPPX Cells Show Increased Antioxidant Levels

**Figure 3:**
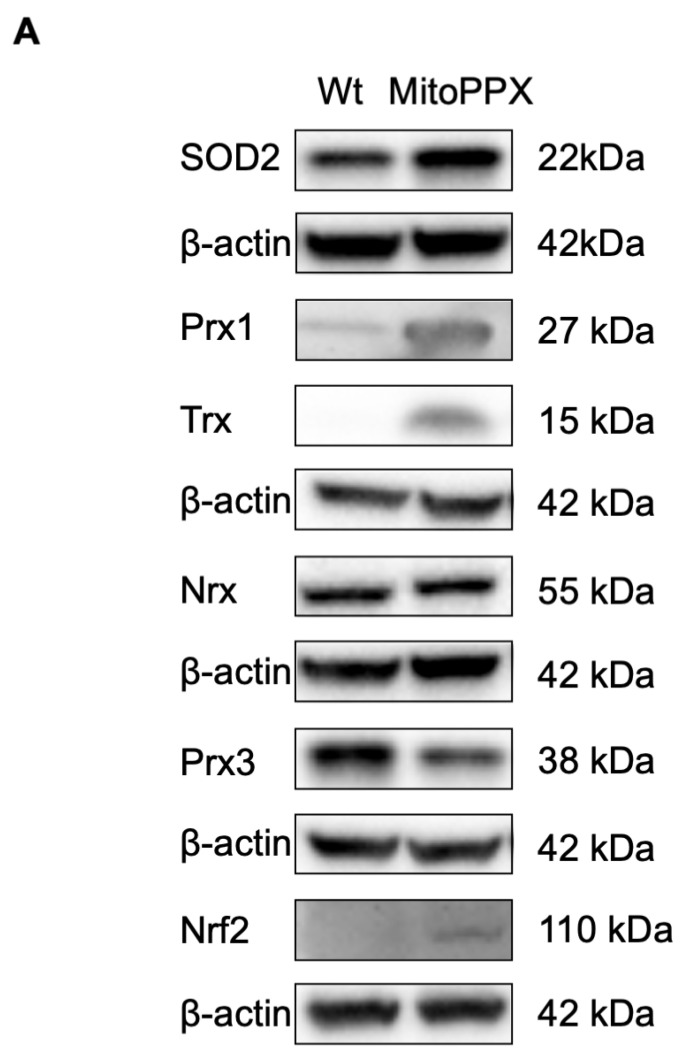

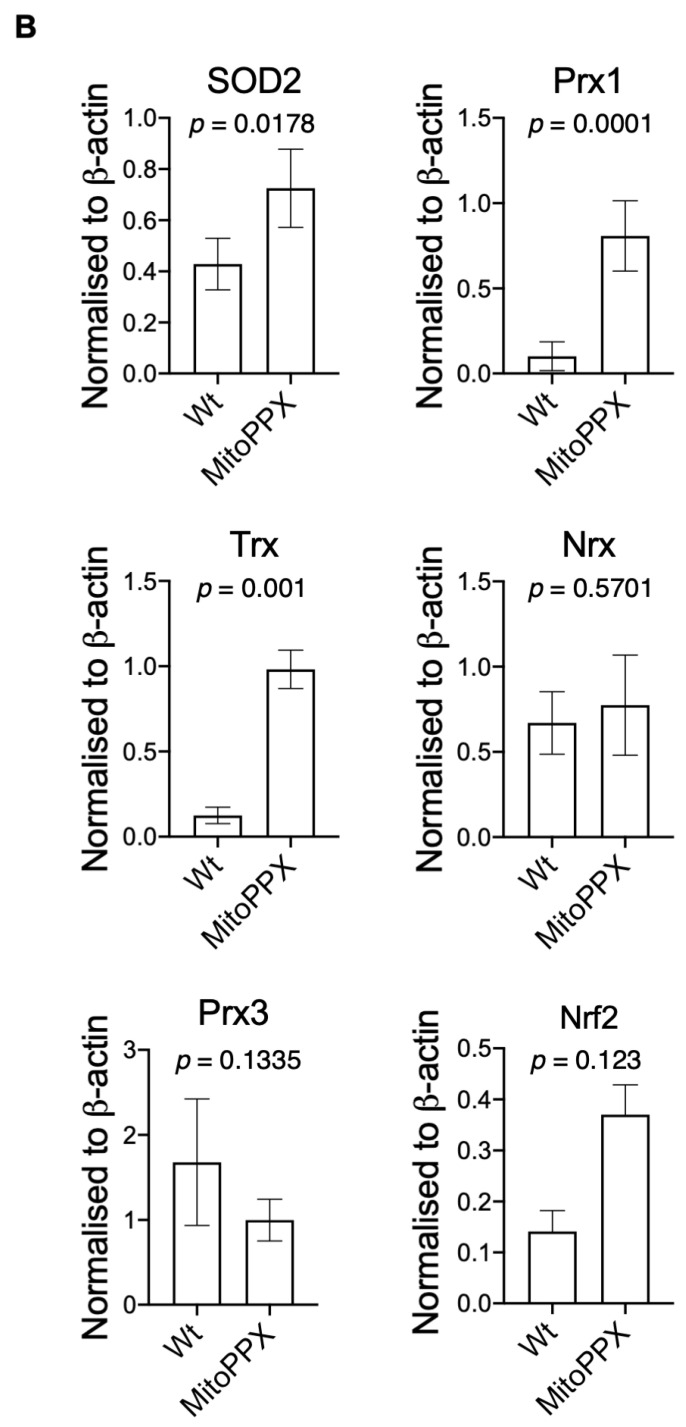
Antioxidant levels are increased in MitoPPX cells. (**A**) Non-reducing immunoblotting was performed to detect the basal levels of SOD2, Prx1, Trx, Nrx, Prx3, and Nrf2 in MitoPPX and Wt cells. Representative membranes are shown. β-actin was used as a loading control. (**B**) Densitometric analysis of basal SOD2, Prx1, Trx, Nrx, Prx3, and Nrf2 protein levels. Data shown as mean ± standard deviation from three independent experiments are presented. Unpaired t-tests were used to detect significant differences between Wt and MitoPPX cells. *Abbreviations used in this figure*: SOD: superoxide dismutase 2; Prx1: peroxiredoxine1; Trx: thioredoxin; Nrx: nucleoredoxin; Prx3: peroxiredoxine 3; Nrf2: nuclear factor erythroid 2-related factor 2; MitoPPX: cells expressing the exopolyphosphatase enzyme on mitochondria; Wt: Wild-type cells.

A typical consequence of the increased presence of ROS is the upregulation and activation of the intracellular antioxidant systems [50,64,65]. Here, we conducted a study of the levels of antioxidants involved in O_2_^−^ and H_2_O_2_ detoxification in our cellular samples by immunoblotting. We observed a significant increase in the protein levels of superoxide dismutase 2 (SOD2), peroxiredoxin 1 (Prx1), thioredoxin (Trx), and nuclear factor-erythroid 2-related factor 2 (Nrf2) in MitoPPX cells when compared to Wt cells. However, the protein levels of peroxiredoxin 3 (Prx3) and nucleoredoxin (Nrx) showed no significant differences between Wt and MitoPPX HEK293 cells (Figure 3).

### 3.4. MitoPPX Cells Show Increased Activation of the PPP

**Figure 4:**
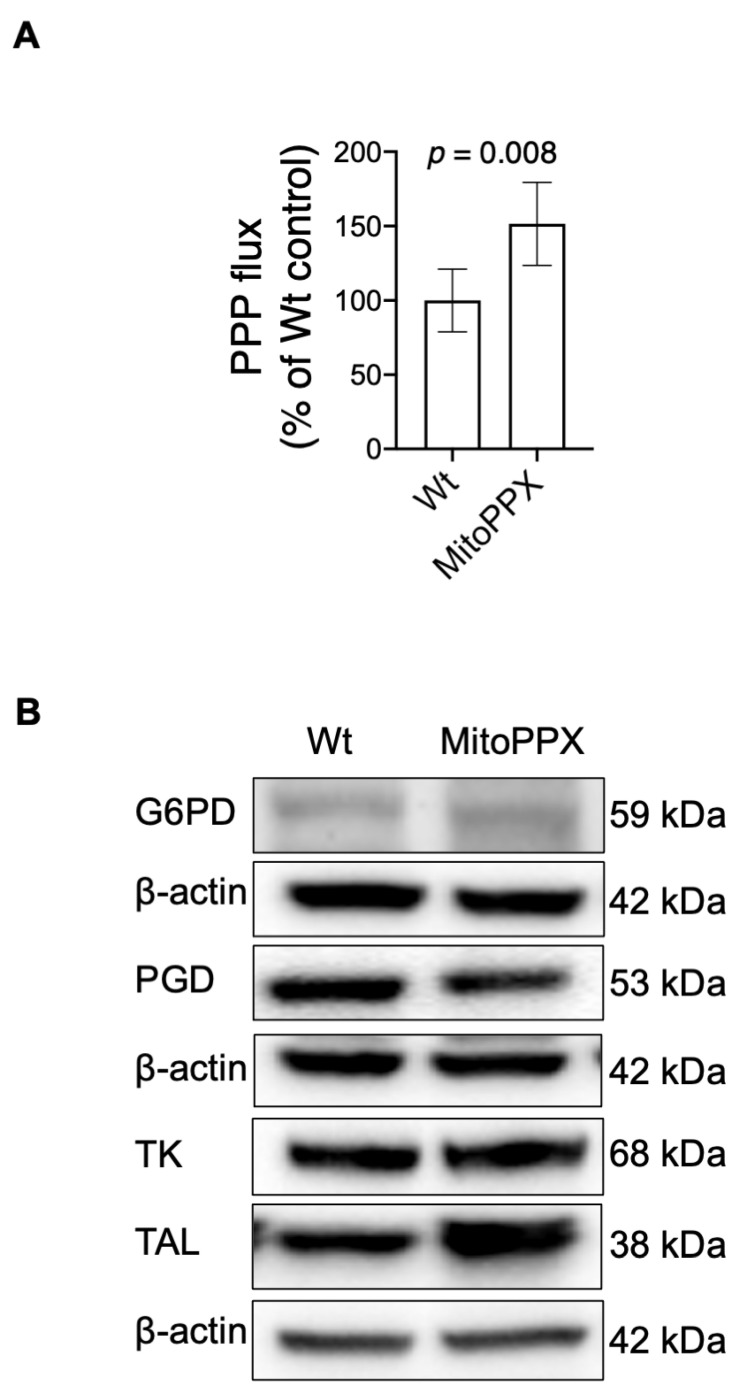

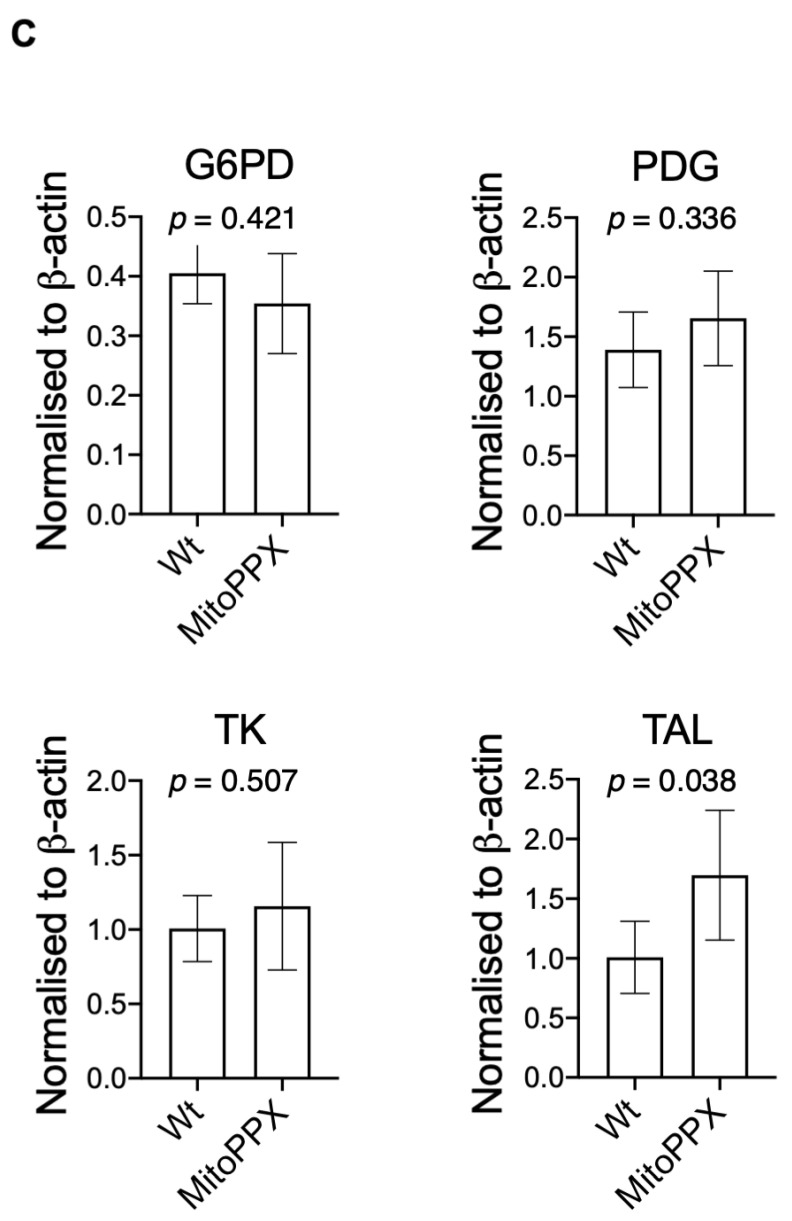
Mitochondrial depletion of polyP increases the protein levels of TAL. (**A**) Graph showing the percentage of glucose flux through the PPP, measured using NMR, in Wt and MitoPPX cells. Data in all graphs are shown as average ± standard deviation of four independent NMR experiments, which were conducted with samples obtained on four separate days. Unpaired t-tests were used to detect significant differences between Wt and MitoPPX cells. (**B**) Non-reducing immunoblotting was performed to detect the basal levels of G6PD and PGD (oxidative phase of the PPP) and TK and TAL (non-oxidative phase of the PPP) in Wt and MitoPPX cells. Representative immunoblots are shown. β-actin was used as a loading control. (**C**) Densitometric analysis of basal G6PD, PGD, TK, and TAL protein levels. Data shown as mean ± standard deviation from *n* = 3 independent experiments are presented. Unpaired t-tests were used to detect significant differences between Wt and MitoPPX cells. *Abbreviations used in this figure:* TAL: transaldolase; PPP: pentose phosphate pathway; NMR: nuclear magnetic resonance; G6PD: glucose-6-phosphate dehydrogenase; PGD: phosphogluconate dehydrogenase; TK: transketolase; MitoPPX: cells expressing the exopolyphosphatase enzyme in mitochondria; Wt: Wild-type cells.

Glycolysis and the PPP are well-known extra-mitochondrial components of mammalian bioenergetics that contribute to maintaining appropriate intracellular bioenergetic status. Here, using ^13^C-NMR spectroscopy, we measured the percentage of glucose flux that was directed specifically towards glycolysis or the PPP in MitoPPX and Wt cells. Our data show a ≅50% increase in the PPP in MitoPPX cells when compared with the Wt samples (*p* ≤ 0.05. Figure 4A). In addition, glycolysis trended towards an increase in MitoPPX cells, which aligns with our previous observations in the same cell lines [17] (Supplementary Figures S3 and S4). These results demonstrate that the enzymatical depletion of mitochondrial polyP produces a global effect on mammalian cellular bioenergetics, not only affecting mitochondrial OXPHOS but also the cytosolic pathways, such as PPP.

### 3.5. The Enzymatic Depletion of Mitochondrial polyP Increases the Protein Levels of TAL

The PPP is a parallel pathway to glycolysis in the metabolism of glucose. The main enzymes involved in this pathway are glucose-6-phosphate dehydrogenase (G6PD) and 6-phosphoglutanate dehydrogenase (PGD) in the oxidative phase, along with transketolase (TK) and transaldolase (TAL) in the non-oxidative phase.

To further analyze the status of the PPP pathway in our samples and corroborate the data obtained by NMR showing an upregulation in the PPP, we analyzed the levels of the above-mentioned enzymes by immunoblotting assays. While we observed no differences between Wt and MitoPPX cells in the protein levels of G6PD, PGD, and TK, a significant increase in the levels of TAL was evident in MitoPPX cells when compared to Wt samples (*p* = 0.038; Figure 4B,C).

## 4. Discussion

Emerging evidence suggests that polyP is a crucial regulator of cellular bioenergetics and redox balance. However, little is known about these effects on mammalian cells. Here, we demonstrate that the enzymatic depletion of mitochondrial polyP increases oxidative stress levels in HEK293 cells. This rise is characterized by a significantly increased presence of O_2_^−^ and H_2_O_2_, which can be explained either by a rise in the generation of these ROS or by a decrease in the mechanisms in charge of removing ROS in MitoPPX cells. Our finding aligns with the research of others, who showed elevated oxidative stress in bacteria lacking polyP [2,8]. Moreover, this effect was associated with a significant increase in the protein levels of the antioxidants SOD2, Prx1, and Trx. The bibliography also shows that increased generation of ROS is often associated with a rise in the levels of antioxidants [72]. Additionally, using NMR spectroscopy, we show that the enzymatical depletion of mitochondrial polyP increases the activity of glycolysis, which aligns with our prior data [17] as well as the activity of the PPP. Of interest is the finding that the increase in the PPP is of a higher magnitude than that of glycolysis. Immunoblotting results indicate that the protein levels of TAL, a crucial enzyme in the non-oxidative branch of the PPP [73], which also plays an important role in the regulation of cellular ROS [56], are increased in MitoPPX HEK293 cells when compared to Wt controls.

Dysfunctional OXPHOS is well regarded as a key mechanism that increases ROS generation and oxidative stress conditions in a wide range of human pathologies [60,74]. Our prior data demonstrate that MitoPPX cells, which have a mitochondrial membrane potential comparable to Wt cells, are sensitive to FCCP treatment and do not show increased levels of mitophagy, display a significant reduction in OXPHOS in MitoPPX cells when compared to Wt cells [17]. This reduction in OXPHOS could be a consequence of mitochondrial dysfunction at the level of the electron transport chain and it likely explains the increased levels of O_2_^−^ and H_2_O_2_ shown in this study. In fact, reduced OXPHOS has been demonstrated to be a trigger of increased levels of ROS [75,76]. For example, it is known that inhibition of Complex I and III of the ETC induces increased chronic generation of H_2_O_2_ in HEK293 cells without substantially increasing the presence of oxidative stress and cell death [77]. Our data also show that elevated ROS levels are associated with a significant increase in the protein levels of mitochondrially-localized SOD2, as well as an increase in the antioxidant transcription factor Nrf2 and the Nrf2-regulated antioxidants Prx1 and Trx. These data suggest that the rise in ROS was sufficient to elicit a cellular response (upregulated antioxidant status) which may have mitigated cell death in this study. As mentioned above, several studies have demonstrated the effect of OXPHOS inhibition to increase the production of mitochondrially derived ROS [75,76,77]. This effect has been shown to increase the levels of mitochondrial SOD2, potentially as a compensatory response to enhance O_2_^−^ detoxification [75,78,79,80]. O_2_^−^ would then be dismutated to form H_2_O_2_, which is now regarded as a potent cell signaling mediator capable of regulating diverse cellular processes [81,82,83,84,85]. One such process is an increase in Nrf2 levels and activity, which in turn leads to an increase in Nrf2-regulated proteins, which include Prx1 and Trx [86,87]. Thus, we postulate that in cells lacking mitochondrial polyP, mitochondrial dysfunction leads to an increase in ROS levels that serve as a signaling molecule that increases Nrf2 and Nrf2-regulated antioxidant proteins. The observed increase in antioxidant protein levels is consistent with our previous research, which shows that MitoPPX cells display a significant increase in reduced glutathione (mean ± stdev of the log2 abundance of reduced glutathione in MitoPPX cells was 25.43 ± 0.35, while in Wt this was 24.50 ± 0.27. *p* = 0.02), as well as increased levels of NADPH when compared to Wt cells [17]. In the same manuscript, all the raw data, including the levels of NADP and other related metabolites, are included.

In mammalian cells, NADPH is mostly produced in the PPP [88]. This led us to hypothesize that the PPP might be regulated by the levels of polyP. In fact, the role of short chain polyP (as a component of the xylulose-5-phosphate) in the regulation of PPP has already been demonstrated in bacteria [89]. Therefore, we analyzed the PPP in response to the enzymatic depletion of mitochondrial polyP, and we observed a significant increase in the activity of this pathway measured by NMR technology in MitoPPX cells. While glycolysis is also increased in MitoPPX cells, the increase in the PPP in relationship with that of glycolysis is of a higher magnitude. These data build on our prior findings showing that mitochondrial polyP depletion not only inhibits OXPHOS and increases glycolysis but also leads to a significant increase in PPP activity. This further demonstrates the importance of polyP on the maintenance of bioenergetic pathway homeostasis. Such a metabolic switch is of significant importance as under pathological conditions, such as Alzheimer’s disease and Parkinson’s disease; OXPHOS is dysregulated and the PPP is increased as a consequence of a rise in the levels of oxidative stress [57,59]. Therefore, MitoPPX cells could be used as a model of these pathologies to study bioenergetic metabolism, as our results suggest that maintenance of PolyP may be integral to maintaining homeostatic bioenergetics and redox homeostasis. In addition, our findings of increased PPP activity in MitoPPX cells could represent an important compensatory cellular effect, aimed at increasing the production of ATP when OXPHOS is dysfunctional or inhibited.

Our data also show that the metabolic enzyme TAL is significantly increased in MitoPPX cells when compared with Wt samples. This suggests that the upregulation of PPP activity in response to mitochondrial polyP depletion could be mediated through TAL, which is a key rate-limiting enzyme in the non-oxidative branch of the PPP [90,91] and is also critical in the regulation of metabolic flux through the PPP [73]. Furthermore, previous studies have demonstrated the involvement of TAL in the production of NADPH in the PPP [73]. Lastly, the overexpression of TAL has been already demonstrated as an extremely powerful tool to increase the flux towards the PPP [39].

In summary, our findings show that the enzymatic depletion of mitochondrial polyP significantly increases the levels of ROS and mitochondrial and cytosolic antioxidants in mammalian cells. Moreover, the PPP displays enhanced activation in these samples, which is associated with an increase in the protein levels of TAL. These results, alongside our previous work, suggest that polyP significantly regulates cellular redox balance and bioenergetics. Consequently, our findings could pave the way towards the development of new pharmacological strategies that target mitochondrial polyP in disease states where dysregulated bioenergetics and increased oxidative stress conditions are observed, such as neurodegenerative disorders, many types of cancers, and diabetes [81,92,93,94,95,96,97,98,99].

## Figures and Tables

**Table 1:** Effects of polyP on cellular physiology. Schematic representation containing some of the main described effects of polyP on cellular physiology. Many of these affects are closely related to the status of cellular bioenergetics in mammalian organisms.

Effects of polyP on Cellular Physiology	
*Effect*	*References*
Bioenergetic buffer and/or direct regulation of bioenergetics	[3,9,10,11,12,13,14,15,16,17,18,20]
Molecular chaperone	[21,22,23,24,25]
Structural component and/or regulator of the mitochondrial permeability transition pore	[12,26,27,28,29,30]
Regulator of mitochondrial calcium homeostasis	[9,10]
Stress response	[2,8,31]

## Data Availability

Data is contained within the article and Supplementary Materials.

## Supplementary Materials

The following are available online at https://www.mdpi.com/article/10.3390/antiox11040685/s1, Figure S1: Corroborating our previous data, MitoPPX cells show decreased levels of ATP and increased glycolysis, when compared with the Wt cells. Figure S2: Characterization of the HEK293 MitoPPX cells. Figure S3: NMR Spectra. Figure S4: NMR Spectra.
