# Supplementary material for: Enzymatic Depletion of Mitochondrial Inorganic Polyphosphate (polyP) Increases the Generation of Reactive Oxygen Species (ROS) and the Activity of the Pentose Phosphate Pathway (PPP) in Mammalian Cells
Source: Antioxidants (Basel). 2022 Mar 31;11(4):685. doi: 10.3390/antiox11040685 (PMC9029763; doi:10.3390/antiox11040685)
Supplement: Supplementary file 1 [file antioxidants-11-00685-s001.zip › antioxidants-1633178-supplementary.pdf]

Supplementary Figures

Enzymatic depletion of mitochondrial inorganic polyphosphate (polyP) increases the generation of reactive oxygen species (ROS), and the activity of the pentose phosphate pathway (PPP) in mammalian cells.

Hambardikar, Vedangi; Guitart-Mampel, Mariona; Scoma, Ernest R.; Urquiza, Pedro; Nagana Gowda, G.A.; Raftery, Daniel; Collins, John A.; & Solesio and Maria E.

Supplementary Figure S1

A

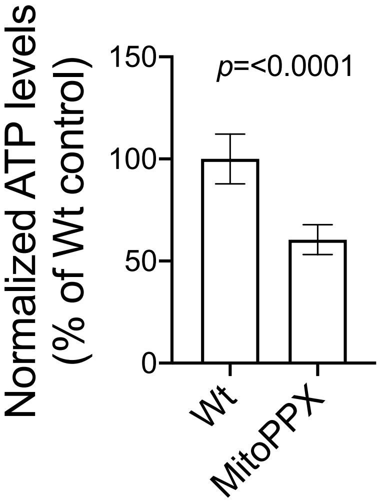

B

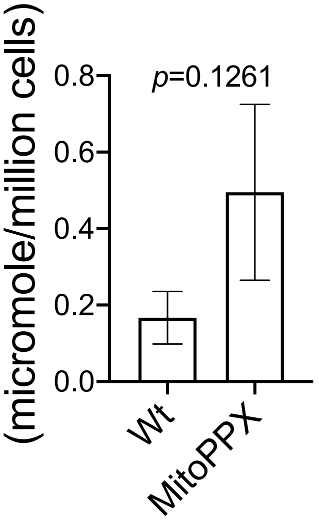

Supplementary Figure S1. Corroborating our previous data, MitoPPX cells show decreased levels of ATP and increased glycolysis, when compared with the Wt cells. **A.** Graph showing the normalized values of ATP in Wt and MitoPPX cells. Note the decreased levels of ATP in MitoPPX HEK293 cells. **B.** Graph showing the concentration of lactate, which is a standard indirect measurement of the activity of glycolysis in mammalian cells, in Wt and MitoPPX samples. Increased levels of glycolysis, assayed by the measurement of the concentration of lactate using NMR, were found in the mutated cells. Data is shown as mean ± SEM from n=4 independent experiments. Unpaired t-tests were used to detect significant differences between Wt and MitoPPX cells.

Supplementary Figure S2

A

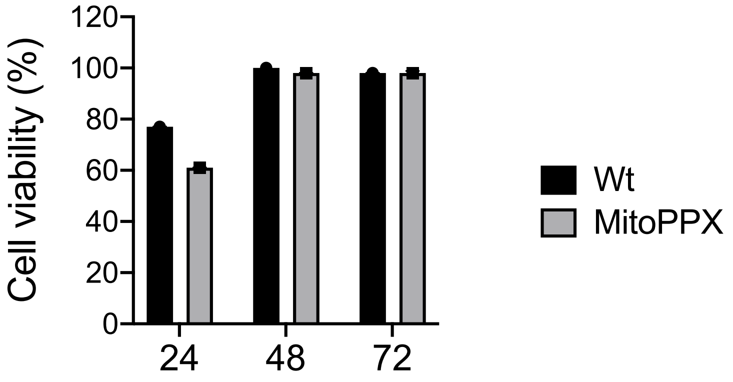

B

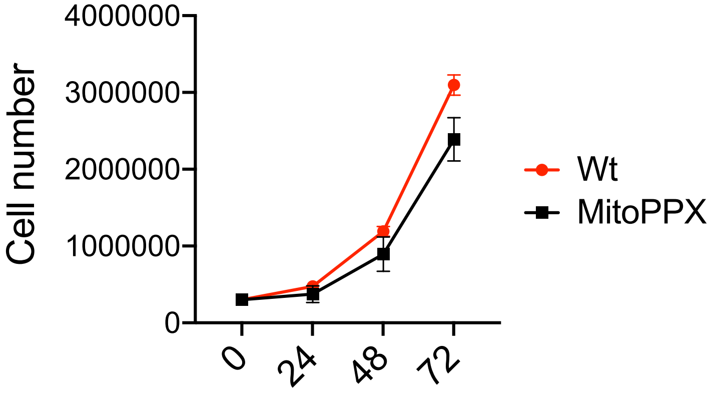

C

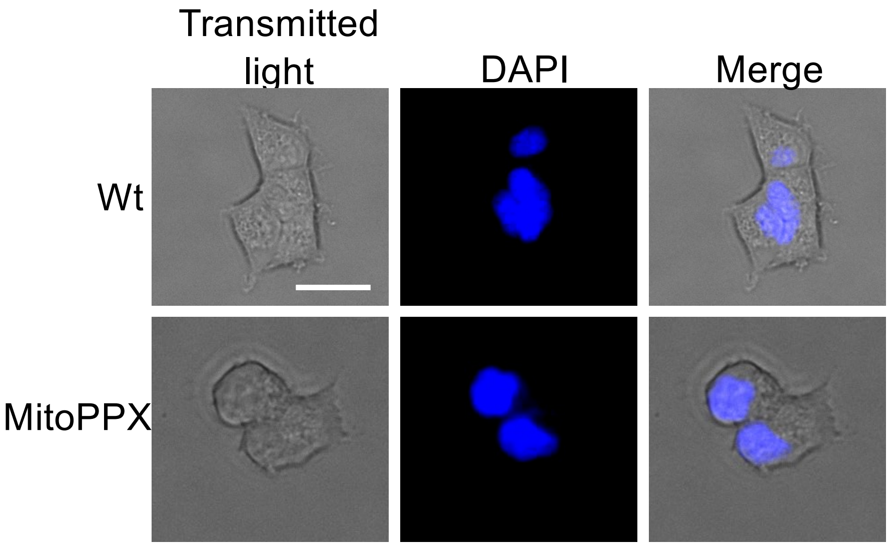

15 μM

Supplementary Figure S2

Supplementary Figure S2. Characterization of the HEK293 MitoPPX cells. **A.** Cell viability assay conducted using trypan blue shows no major differences in this parameter between Wt and MitoPPX cells, 48 hours after seeding them. **B.** Using the same method and time points at 24 hours, 48 hours, and 72 hours, our data also show similar growing curves for Wt and MitoPPX cells. **C.** Representative images obtained using transmitted fluorescence microscopy and DAPI staining for the nuclei show no significant differences in terms of cell size or morphology between Wt and MitoPPX cells. Data is shown as mean ± standard deviation from three independent experiments are presented. Unpaired t-tests were used to detect significant differences between Wt and MitoPPX cells. Scale bar = 15 μM.

Supplementary Figure S3

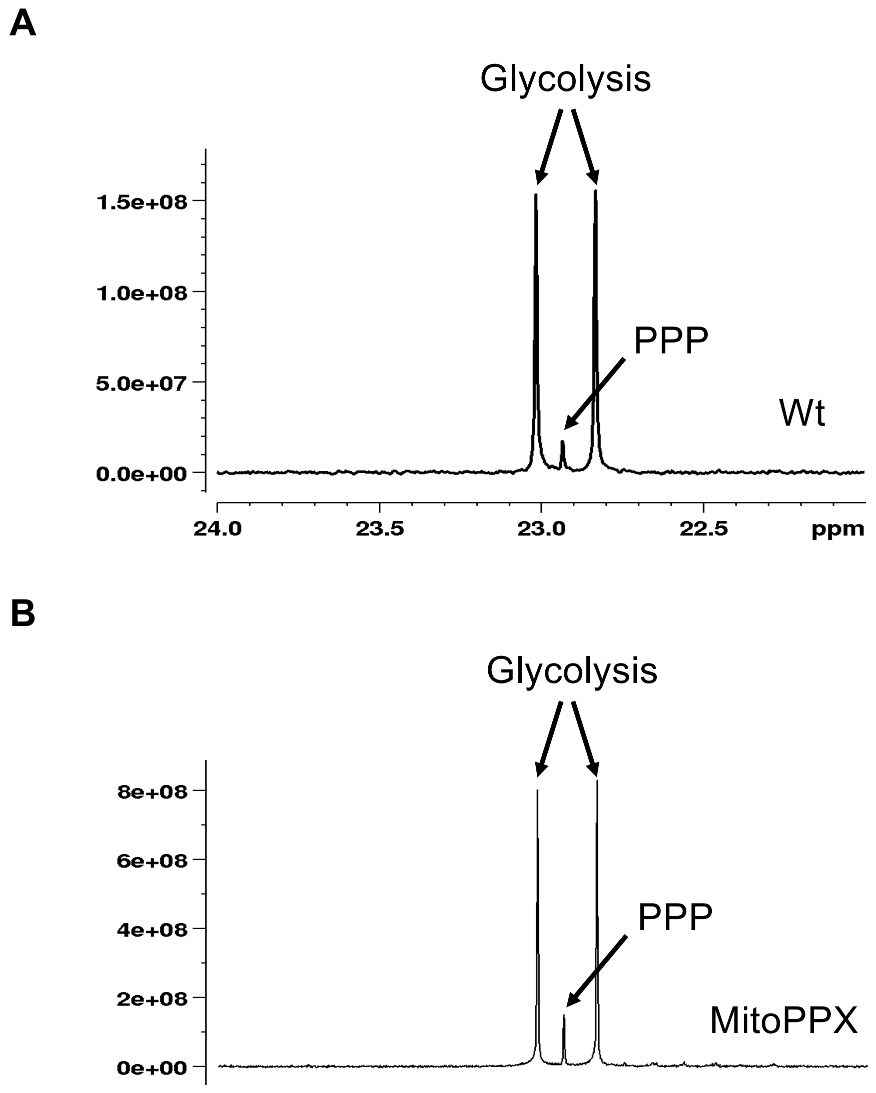

Supplementary Figure S3. NMR Spectra. Parts of typical 800MHz NMR spectra of **A.** Wt and **B.** MitoPPX cells highlighting lactate peaks that arise from glycolysis and the PPP. Total peak areas of lactate in A. and B. are matched for visualization of the PPP in the two cell types. Note that the NMR peak corresponding to the PPP is of a higher intensity in MitoPPX cells when compared with the Wt samples, which indicates higher flux of lactate through the PPP in these cells when compared to the flux through glycolysis.

Supplementary Figure S4

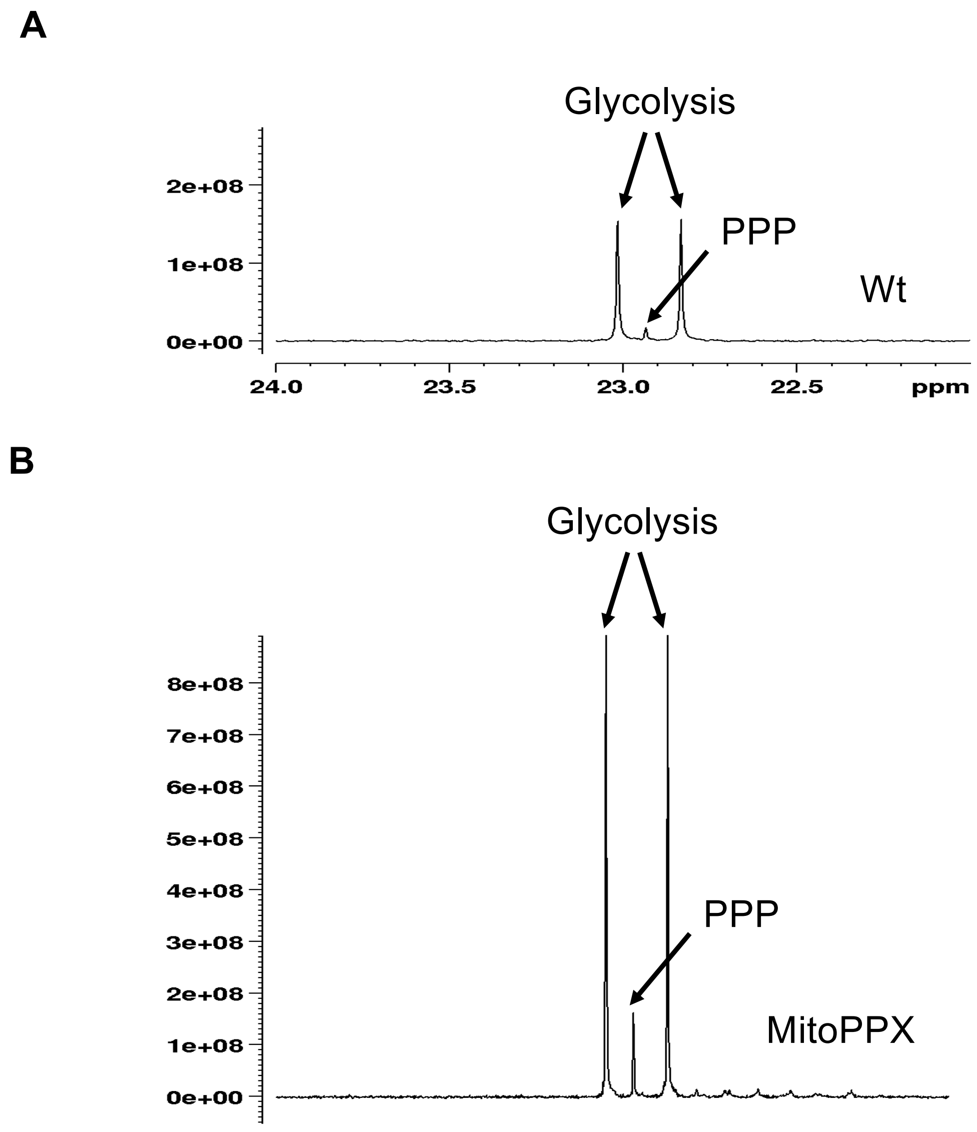

Supplementary Figure S4. NMR Spectra. Parts of typical 800MHz NMR spectra of **A.** Wt and **B.** MitoPPX cells highlighting lactate peaks that arise from glycolysis and the PPP. Peak intensities in A. and B. are normalized based on cell counts. Note the significantly higher magnitude of glycolysis and PPP in the MitoPPX cells when compared to the Wt samples.
